# High Expression of IGSF10 Confers an Inhibitory Effect on the Progression of Lung Adenocarcinoma

**DOI:** 10.1111/jcmm.70995

**Published:** 2025-12-25

**Authors:** Lianyu Cheng, Beibei Ma, Yun Zhao, Chunyan Qin, Lihe Jiang, Bo Ling

**Affiliations:** ^1^ College of Basic Medical Sciences Youjiang Medical University for Nationalities Baise Guangxi China; ^2^ Department of Pulmonary and Critical Care Medicine Affiliated Hospital of Youjiang Medical University for Nationalities Baise Guangxi China; ^3^ Zhejiang Key Laboratory of Diagnosis & Treatment Technology on Thoracic Oncology (Lung and Esophagus), Zhejiang Cancer Hospital Hangzhou China; ^4^ College of Pharmacy, Youjiang Medical University for Nationalities Baise Guangxi China

**Keywords:** cell cycle, IGSF10, lung adenocarcinoma, p53‐p21 axis, therapeutic target

## Abstract

Lung cancer is one of the most frequently diagnosed cancers and the leading cause of cancer‐related deaths worldwide. Unlike conventional treatments, the targeted therapies or emerging immunotherapies have shown significant advantages in the management of advanced lung cancer. Therefore, exploring novel predictive biomarkers or therapeutic targets is still of far‐reaching significance for the future treatment of lung cancer. This study revealed that low expression of IGSF10, an important member of the immunoglobulin superfamily, significantly correlates with poor overall survival of lung adenocarcinoma (LUAD) patients and strong tumorigenic capacity of LUAD cells. Mechanistically, high expression of IGSF10 can inhibit the epithelial‐mesenchymal transition of LUAD cells via p53‐triggering ferroptosis and impede G_1_/S cell cycle transition of LUAD cells via the p53‐p21 axis, leading to suppression of LUAD cell migration, growth and tumorigenic capacity. Our findings clarified the specific role of IGSF10 in LUAD, and theoretically suggested new avenues for the presumable IGSF10‐targeting therapy of lung cancer in the future.

## 1 Introduction

Despite significant progress in elucidating disease pathogenesis, applying predictive biomarkers, and optimising therapeutic strategies in the past, lung cancer remains one of the most frequently diagnosed cancers and the leading cause of cancer‐related deaths worldwide, with an estimated 2.20 million new cases and 1.79 million deaths per year [1]. Histologically, lung cancer is a heterogeneous disease with a wide range of clinicopathological features, which can be broadly divided into non‐small‐cell lung cancer (NSCLC, 85% of total diagnoses) or small‐cell lung cancer (SCLC, 15% of total diagnoses) [2]. Furthermore, lung adenocarcinoma (LUAD) is the most common subtype of NSCLC followed by lung squamous‐cell carcinoma (LUSC), whereas the proportions of them vary according to race [3, 4]. Prior to clinical treatment, adequate staging is crucial for determining the most appropriate therapy to the patients with lung cancer and it often relies on the use of imaging methods, including fluorodeoxyglucose‐PET (FDG‐PET) scans and magnetic resonance imaging (MRI); meanwhile, the bronchoscopic and radiological methods for tissue biopsy samples are also regularly employed [5]. For early‐staged lung cancer, besides the conventional surgical resection, fractionated radiotherapy was considered as an alternative if the patients are medically inoperable [6]. However, the management of advanced lung cancer often involves chemotherapy or targeted therapies based on histology and genetics [1, 5]. Therefore, the discovery of novel predictive biomarkers has provided new therapeutic opportunities for the targeted therapies and/or emerging immunotherapies.

The immunoglobulin (Ig) superfamily (IGSF) is the largest protein superfamily whose members typically comprise an Ig homology (Ig‐like) domain formed by several anti‐parallel β‐sheets stabilised with disulfide bonds [7]. Based on their properties of Ig‐like domains forming rod‐like structures and binding specifically to target proteins, IGSF proteins ideally act as various antibodies, cell surface receptors and cell adhesion molecules (CAMs), functioning in complex biological networks [8]. An important member of IGSF, IGSF10 has been previously shown to implicate diverse developmental processes. For example, *IGSF10* mutations can result in delayed puberty via dysregulating gonadotropin‐releasing hormone (GnRH) neuronal migration and IGSF10 deficiency‐triggered transient GnRH deficiency may lead to a reversible congenital hypogonadotropic hypogonadism (CHH) [9, 10, 11]. Moreover, *IGSF10* mutations have also been viewed as key contributors to other developmental diseases, including combined pituitary hormone deficiencies (CPHD), cleidocranial dysplasia (CCD), and Kallmann syndrome (KS) [12, 13, 14]. Nowadays, the correlation between IGSF10 and cancers is becoming increasingly attractive. A recent systematic pan‐cancer analysis of *IGSF10* based on multiple databases has concluded that *IGSF10* can serve as a valuable prognostic biomarker for certain types of cancers albeit its aberrant expression across different tumours, and *IGSF10* expression was closely correlated with the tumour‐infiltrating immune cells, immune checkpoints, and immune modulators, suggesting IGSF10 as a potential immunotherapy target for several malignancies [15].

Over the past few years, growing proofs have also validated the definitive role of IGSF10 as a prominent biomarker in a variety of cancers, including osteosarcoma [16], endometrial cancer [17], oral tumour [18], and multitype breast cancers [19, 20, 21]. Moreover, genetic mutations of IGSF10 might contribute to a higher risk of rectal and gastric cancers [22]. Nevertheless, there have been few reports on the relevance of IGSF10 to the pathogenesis of lung cancer in the past, although we preliminarily confirmed its involvement in the cellular function of lung cancer [23, 24]. However, the molecular mechanism underlying the modulation of IGSF10 in lung cancer still needs further understanding, and the present study is aiming to explore this important issue.

## 2 Materials and Methods

### 2.1 Dataset Acquisition and Analysis

We downloaded the standardised pan‐cancer dataset TCGA TARGET GTEx (PANCAN, *N* = 19,131, G = 60,499) from the UCSC Xena database (https://xenabrowser.net/). Subsequently, we extracted the expression data of the gene IGSF10 (ENSG00000152580) in each sample and screened out the samples from the TCGA database. To ensure the reliability of subsequent analysis, we further screened samples from different sources and performed log2 (x + 1) transformation on each expression value. Finally, we eliminated cancer types with less than 3 samples and obtained expression data of 26 cancer types for comprehensive analysis of the expression pattern of IGSF10.

### 2.2 IGSF10 Differential Expression and Survival Analysis

Differential expression analysis of tumour tissues and paired adjacent normal tissues was performed using the DESeq2 R package (version 4.2.2). The internal normalisation procedure of this package was used to correct for sequencing depth and RNA composition bias. The screening criteria for differentially expressed genes (DEGs) were adjusted *p*‐value (*p*‐adj) < 0.05 and |log2 fold change (log2 FC)| > 1. Subsequently, we obtained the high‐quality TCGA prognostic dataset from the TCGA prognostic study published in Cell, and obtained the TARGET follow‐up data from the UCSC Xena Cancer Browser (https://xenabrowser.net/datapages/) as a supplement. After excluding samples with a follow‐up time of less than 30 days, all expression values were log2 (x + 1) transformed. To ensure statistical power, we further excluded cancer types with a sample size of less than 10 cases. Finally, we obtained gene expression data and corresponding overall survival data for 44 cancer types. Univariate Cox regression analysis was used to calculate the hazard ratio (HR) and its 95% confidence interval (95% CI). In the Cox proportional hazard model, the IGSF10 low expression group was used as the reference group. HR < 1 indicated that high IGSF10 expression was associated with a significant improvement in prognosis, whereas a negative expression indicated a poor prognosis.

### 2.3 IGSF10 Enrichment Analysis and Single‐Cell Analysis

Based on the median expression level of IGSF10 in lung adenocarcinoma tissues, the samples were divided into IGSF10 high expression group and low expression group. Subsequently, the differentially expressed genes (DEGs) between the two groups were identified, and gene ontology (GO) and gene set enrichment analysis (GSEA) were performed to determine the differentially enriched pathways between the two groups. GSEA analysis was performed on the preselected gene sets using the R package clusterProfiler to compare the differences between the high expression group and the low expression group. The identification of differentially expressed genes was performed using the Limma or edgeR software package. For single‐cell data analysis, NSCLC‐GSE99254 single‐cell data and annotation files were downloaded from the TISCH database, and R software MAESTRO and Seurat were used for data processing and analysis, and the t‐SNE method was used to re‐cluster and group the cells.

### 2.4 Cell Culture and Generation of Stably Overexpressed Cell Lines

Human lung adenocarcinoma cell line A549 was cultured in RPMI1640 medium (Gibco, USA), supplemented with 10% fetal bovine serum (Gibco, USA), 100 U/mL penicillin (Thermo Fisher Scientific, USA), 0.1 mg/mL streptomycin (Thermo, USA), and kept within the 37°C incubator under 5% CO_2_. Human Non‐small cell lung cancer cell line H1299 was cultured in RPMI1640 medium (Gibco, USA), supplemented with 10% fetal bovine serum (Gibco, USA), 100 U/mL penicillin (Thermo Fisher Scientific, USA), 0.1 mg/mL streptomycin (Thermo, USA), and kept within the 37°C incubator under 5% CO_2_. Lentiviral constructs (Flag‐IGSF10 and GFP‐IGSF10) were generated by PCR‐based cloning method and confirmed by DNA sequencing and packaged using HEK293 cells; the control cells were accordingly transfected with empty vector. The shRNA sequence for IGSF10 is as follows: 5′‐CCCAATGTGGAACGCATCAAT‐3′.

### 2.5 Western Blot

Cells were lysed using 1× SDS lysis buffer and analysed by SDS‐PAGE with the antibodies as follows: IGSF10 (ab166199, Abcam, UK), p53 (ab1101), p21 (ab227443), Snail (ab216347), Slug (ab302780), E‐cadherin (ab231303), SLC7A11 (ab307601), GPX4 (ab252833), β‐actin (Huabio, China). Relative quantitative expressions were calculated in grayscale.

### 2.6 Cell Migration Assay

For the scratch assay, A549 and H1299 cells were seeded and scratched with micropipette tips when the cells had grown to a confluency of near 90% and the images were captured at 0, 12, 24, and 48 h after wounding. Transwell chambers (8 mm pore size; MilliporeSigma) were used according to the manufacturer's instruction and the number of cells in three random fields on the underside of the filter was counted. Data represent three independent experiments performed in duplicate.

### 2.7 Colony Formation Assay

Approximately 300 cells were seeded into 6‐well plate with pre‐warmed 1640 and incubated for 2 weeks. Next, the cells were gently washed twice with PBS (Sangon Biotech, China), then fixed with 4% PFA (Sangon Biotech, China) for 30 min and stained with Crystal Violet Staining Solution (Sangon Biotech, China) for 2 h. Then, the colonies were photographed and analysed.

### 2.8 Cell Apoptosis and Cell Cycle Assay

Cells were harvested and stained with the *Annexin V Apoptosis Detection Kit* (Elabscience, China), and the percentage of Annexin V‐positive cells was detected by FACSCalibur flow cytometry (BD Biosciences, USA). Cells were seeded into 6‐well plates and harvested overnight, then stained with Cell Cycle Assay Kit (Elabscience, China) for 30 min. Harvested cells were fixed in ice‐cold 70% ethanol for 2 h, and rinsed with PBS. Cells were incubated with prepared buffer (PBS 438 mL, CuSO_4_ 10 mL, fluorescent dye azide 2.5 mL, 1× reaction buffer 50 mL) and resuspended in PBS containing RNase A (100 mg/mL), then stained with PI Reagent (50 μg/mL), and analysed by flow cytometry. Percentage of the indicated cell phases was analysed using FlowJo software.

### 2.9 Tumour Formation Assay

The nude mice aged 4–6 weeks were purchased from Beijing Weitong Lihua Experiment Animal Technology Co (Beijing, China). 5 × 10^6^ IGSF10‐OE and/or sh‐IGSF10 A549 cells and the control cells were injected into the dorsal flanks of mice subcutaneously, respectively. Eight weeks after injection, tumours were separated from mice and photographed and weighed. All procedures were performed in accordance with the guidelines for the care and use of laboratory animals of the Youjiang Medical University for Nationalities Institutional Review Board.

### 2.10 ROS Assay

Cells were harvested and stained with the ROS assay kit (Beyotime, China), and the cells were analysed using a BD FACSCalibur flow cytometry (BD Biosciences, USA).

### 2.11 Lipid Peroxidation Assay

The cells in 6‐well plates were collected and fixed with 1 mL BODIPY 581/591C11 staining solution, incubated at 37°C for 30 min. Harvested cells were rinsed with PBS and observed under a fluorescence microscope.

### 2.12 Detection of Iron

Cells were grown in 6‐well plates, washed twice with PBS and incubated with Cell Iron Content Assay Kit (Solarbio, China). Stained cells were measured at 510 nm with a microplate reader (Thermofish, USA).

### 2.13 Statistical Analysis

Statistical analysis and graphics were performed using *GraphPad Prism* software. Statistical significance was calculated by two‐tailed Student's t‐tests and error bars represented the S.D., *p < 0.05, **p < 0.01, ***p < 0.001, ****p < 0.0001.

## 3 Results

### 3.1 Downregulated Expression of IGSF10 in Multiple Cancer Types and Its Characteristic Distribution at the Tissue and Single‐Cell Levels

**FIGURE 1:**
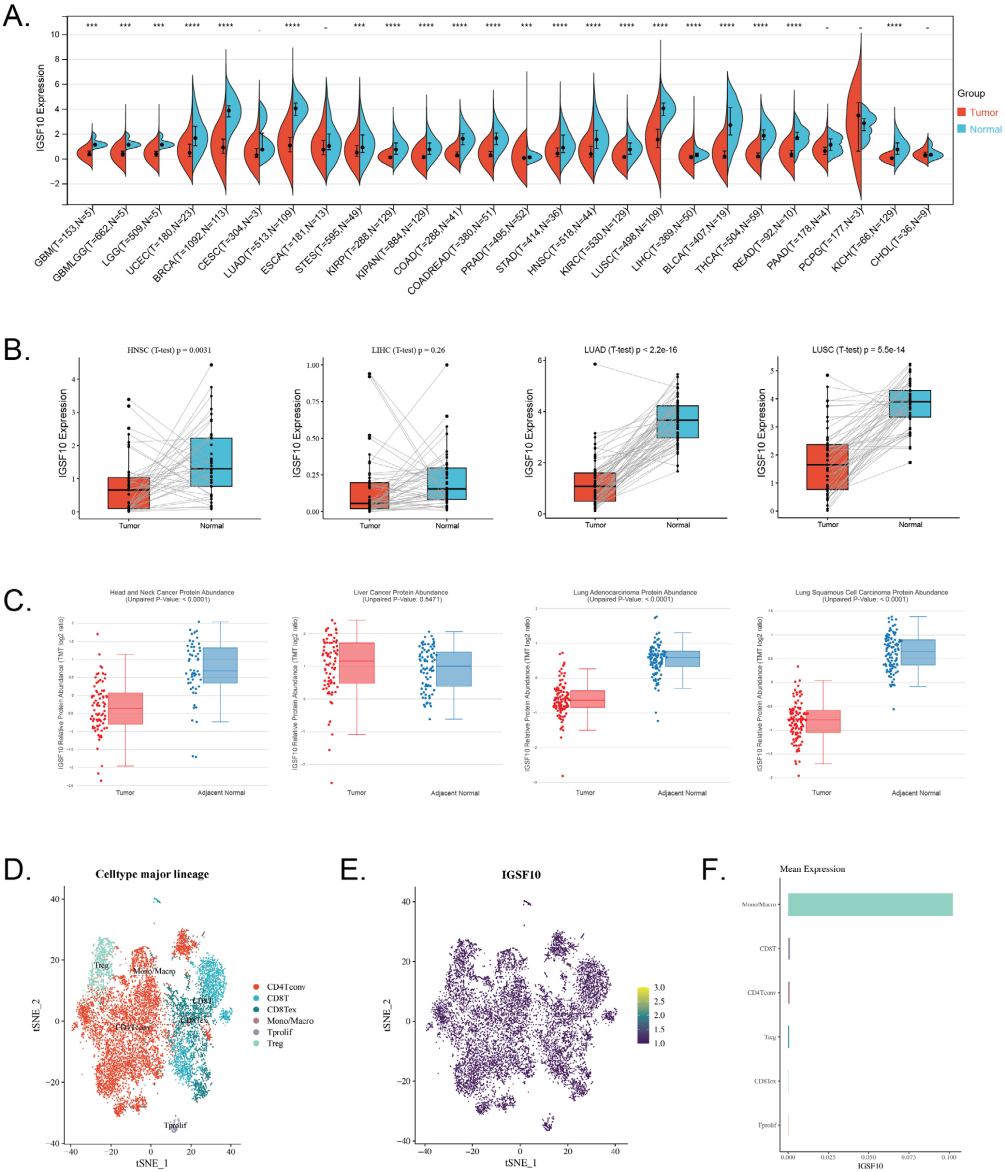
Expression profile and expression level of IGSF10 in different cancers. (A) Differential expression analysis of IGSF10 between tumour tissues and corresponding normal tissues. *p < 0.05, **p < 0.01, ***p < 0.001, ****p < 0.0001. (B) Comparison of IGSF10 expression levels in tumours and adjacent noncancerous tissues from the same patient. (C) IGSF10 protein expression levels in tumours and adjacent non‐cancerous tissues. (D) Single‐cell clustering t‐SNE diagram, different colours represent different types of cells. (E) t‐SNE diagram of the expression distribution of IGSF10 in different cells. Different colours represent the expression abundance. The darker the colour, the lower the expression of the gene in the cell, and the brighter the colour, the higher the expression of the gene in the cell. (F) Histogram of IGSF10 expression abundance in different cells.

We used R software to calculate the expression difference between normal samples and tumour samples in each tumour, and used unpaired Wilcoxon Rank Sum and Signed Rank Tests for difference significance analysis. We observed significant downregulation of IGSF10 in 21 tumours, such as UCEC (Tumour: 0.75 ± 0.79, Normal: 1.94 ± 1.16, *p* = 1.2e‐7), BRCA (Tumour: 1.09 ± 0.84, Normal: 3.73 ± 0.79, *p* = 1.9e‐62), LUAD (Tumour: 1.27 ± 0.95, Normal: 3.99 ± 0.68, *p =* 3.6e‐55), STAD (Tumour: 0.62 ± 0.65, Normal: 1.17 ± 0.87, *p =* 4.7e‐5), HNSC (Tumour: 0.71 ± 0.83, Normal: 1.65 ± 1.01, *p =* 2.9e‐11), LUSC (Tumour: 1.70 ± 1.08, Normal: 3.99 ± 0.68, *p =* 1.5e‐49), etc. (Figure 1A). In addition, we analysed the differential expression of the IGSF10 gene in cancer tissue and adjacent tissue of the same patient to determine that IGSF10 was significantly underexpressed in cancer tissue (Figure 1B). The expression of proteins in cancer tissue and adjacent tissue was analysed using the cProSite database, and the results showed that IGSF10 was significantly underexpressed in HNSC, LUAD, and LUSC, while the opposite was true in LIHC (Figure 1C). We downloaded the single‐cell data of NSCLC‐GSE99254, clustered the cells, and drew a t‐SNE diagram, which showed the expression distribution of IGSF10 in different cells. Different colours represent expression abundance. The darker the colour, the lower the expression of the gene in the cell, and the brighter the colour, the higher the expression of the gene in the cell. The bar graph showed that IGSF10 was more abundant in Mono/Macro (Figure 1D–F).

### 3.2 The Prognostic Value of IGSF10 in Pan‐Cancer and Its Anti‐Cancer Effect in Lung Adenocarcinoma

**FIGURE 2:**
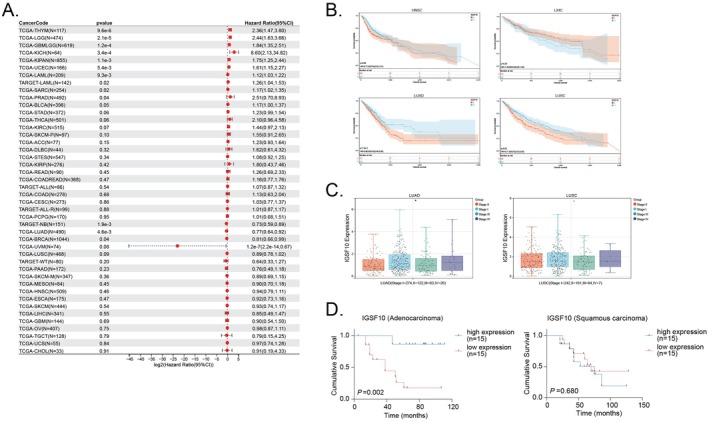
Relationship between IGSF10 expression and prognosis of various cancers. (A) Cox proportional hazards model for the correlation between IGSF10 expression and overall survival (OS). (B) Kaplan–Meier analysis of the association between IGSF10 expression and overall survival. (C) Differential expression of IGSF10 in samples of different clinical stages. *p < 0.05. (D) Kaplan–Meier analysis of the relationship between IGSF10 expression and overall survival in lung cancer patients at local hospitals.

A systematic pan‐cancer analysis using multiple bioinformatics ways has shown differential expression of *IGSF10* in normal and tumour tissues, indicating the prognostic value of IGSF10 in human pan‐cancer [15]. In addition, we established a Cox proportional hazards regression model based on the coxph function of the R software package survival (version 3.2‐7) to analyse the relationship between gene expression and prognosis in each tumour. Statistical tests were performed using the Log‐rank test to obtain prognostic significance. Through the forest plot, high expression of IGSF10 was observed to be associated with poor prognosis in 10 tumour types (TCGA‐GBMLGG (*N* = 619, *p =* 1.2e‐4, HR = 1.84 (1.35, 2.51)), TCGA‐LGG (*N* = 474, *p =* 2.1e‐5, HR = 2.44 (1.63, 3.66)), TCGA‐UCEC (*N* = 166, *p =* 5.4e‐3, HR = 1.61 (1.15, 2.27)), TARGET‐LAML (*N* = 142, *p =* 0.02, HR = 1.26 (1.04, 1.53)), TCGA‐SARC (*N* = 254, *p =* 0.02, HR = 1.17 (1.02, 1.35)), TCGA‐KIPAN (*N* = 855, *p =* 1.1e‐3, HR = 1.75 (1.25, 2.44)), TCGA‐PRAD (*N* = 492, *p =* 0.04, HR = 2.51 (0.70, 8.93)), TCGA‐THYM (*N* = 117, *p =* 9.6e‐6, HR = 2.36 (1.47, 3.80)), TCGA‐LAML (*N* = 209, *p =* 9.3e‐3, HR = 1.12 (1.03, 1.22)), TCGA‐KICH (*N* = 64, *p =* 3.4e‐4, HR = 8.60 (2.13, 34.82))), while low expression of IGSF10 was associated with poor prognosis in 3 tumour types (TCGA‐BRCA (*N* = 1044, *p =* 0.04, HR = 0.81 (0.66, 0.99)), TCGA‐LUAD (*N* = 490, *p =* 4.6e‐3, HR = 0.77 (0.64, 0.92)), TARGET‐NB (*N* = 151, *p* = 1.9e‐3, HR = 0.73 (0.59, 0.89))) (Figure 2A). We further divided the patients into two groups based on the expression level of IGSF10 (H represents the high expression group and L represents the low expression group). Figure 2B shows the Kaplan–Meier survival curves for the four cancer types. By comparing their differences in overall survival (OS), we found that low expression of IGSF10 was significantly associated with poor prognosis in LUAD, LUSC and HNSC, but had no significant prognostic significance in LIHC. In addition, we calculated the expression difference of genes in each tumour in samples of different clinical stages, used unpaired Student's *t*‐test for pairwise difference significance analysis, and used analysis of variance for difference test of multiple groups of samples. We observed that LUAD (Stage I = 274, II = 122, III = 83, IV = 26) (*p* < 0.05) was significantly associated with clinical stage (Figure 2C). The above results suggest that IGSF10 may play a tumour‐suppressive role in LUAD and is significantly associated with better overall survival in patients. To test our hypothesis, Kaplan–Meier analysed the overall survival (OS) of patients from local hospitals. The results showed that the OS of LUAD patients with low IGSF10 expression was significantly lower than that of patients with high IGSF10 expression (*p* < 0.01, Figure 2D), however, this condition was not met for LUSC.

### 3.3 Expression of IGSF10 in NSCLC Tissues Was Correlated With the Tumorigenicity of LUAD After Knockdown

**FIGURE 3:**
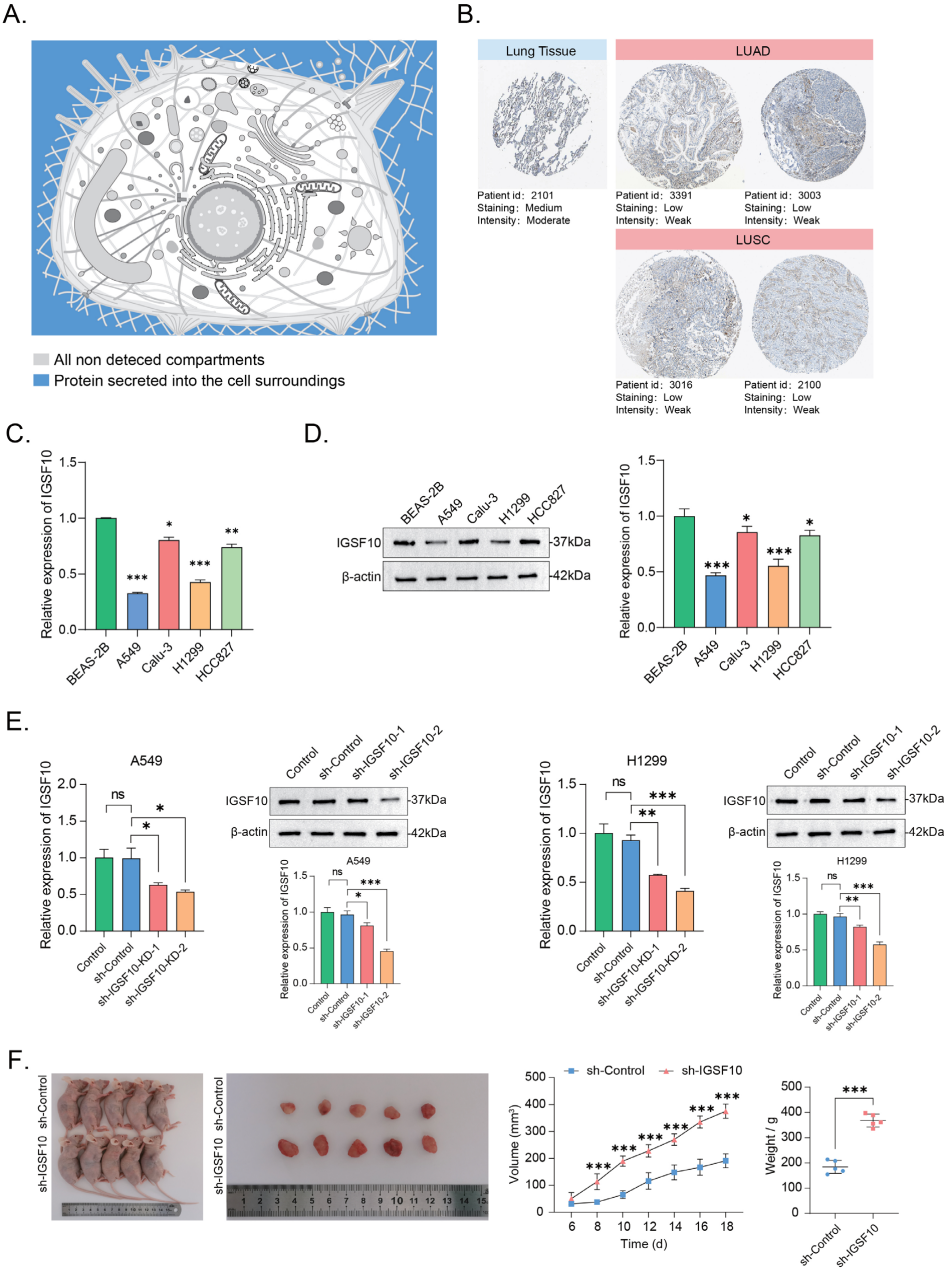
Expression of IGSF10 in NSCLC tissues was correlated with the tumorigenicity of LUAD after knockdown. (A) Subcellular localization profile of IGSF10 in human cells in the UniProt database. (B) IHC analysis of IGSF10 expression in LUAD and LUSC in the HPA database. (C) qRT‐PCR analysis was used to detect the expression of IGSF10 gene in human normal lung epithelial cells and lung adenocarcinoma cells. (D) The expression of IGSF10 protein in human normal lung epithelial cells and lung adenocarcinoma cells was detected by Western Blot analysis. (E) Stable knockdown of Flag‐tagged IGSF10 in human adenocarcinoma cell A549 and H1299 was examined by qRT‐PCR and Western Blot analysis; ns, non‐specific. (F) LUAD cells were infected with lentivirus which code sh‐Control or sh‐IGSF10 and then the cells were injected subcutaneously into the dorsa of nude mice. Tumour weight was measured after 8 weeks. *p < 0.05, **p < 0.01, ***p < 0.001.

We found the expression pattern of IGSF10 protein through the Uniprot database, suggesting that IGSF10 protein is usually secreted outside the cell to exert its effects (Figure 3A). In addition, IHC images of IGSF10 were obtained through the HPA database, and it was found that the expression of IGSF10 was low in LUAD (Figure 2B). We also performed qRT‐PCR and western blotting analysis on normal cells and lung adenocarcinoma cells. From the level of IGSF10 in normal cells and lung adenocarcinoma cells, the expression of IGSF10 in lung adenocarcinoma cells was lower than that in normal cells (Figure 3C,D). Furthermore, an in vivo study on lung tumorigenesis confirmed that knocking down IGSF10 in LUAD cells (Figure 3E) resulted in a significantly enhanced tumorigenicity compared to the control group (Figure 3F). In summary, these findings indicate that low expression of IGSF10 is significantly associated with the incidence of cancer in LUAD patients and that LUAD cells have a stronger tumorigenesis ability (*p* < 0.001).

### 3.4 High Expression of IGSF10 Suppresses LUAD Cell Migration, Growth and Tumorigenic Capacity

**FIGURE 4:**
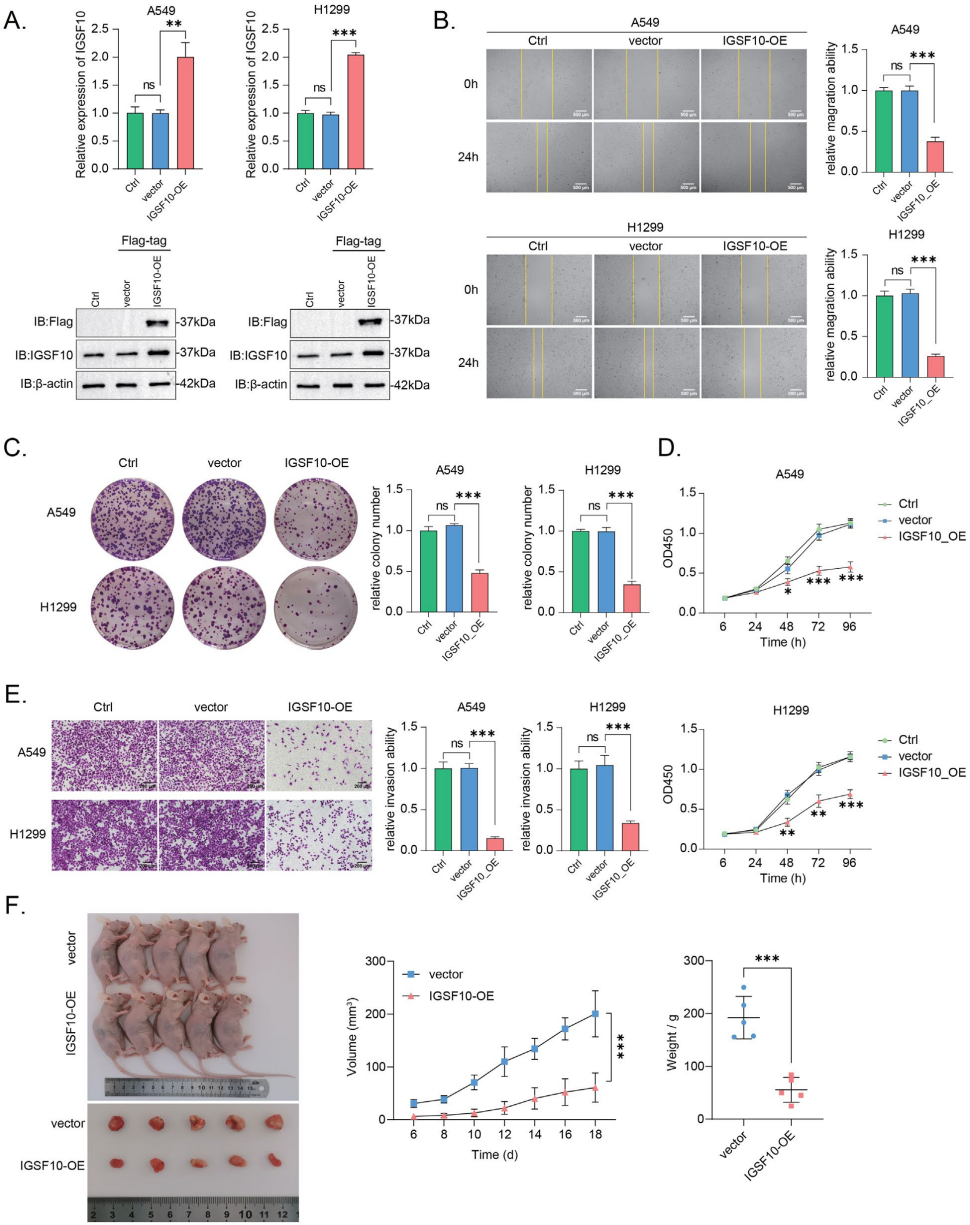
High expression of IGSF10 suppresses LUAD cell migration, growth and tumourigenic capacity. (A) Stable overexpression of Flag‐tagged IGSF10 in human adenocarcinoma cell A549 and H1299 was examined by qRT‐PCR and Western Blot analysis. (B) Overexpressing IGSF10 suppressed A549 and H1299 cells migration measured by scratch assay. (C) Overexpressing IGSF10 suppressed the colony formation of A549 and H1299 cells analysed by colony formation assay. The quantitative histogram was displayed below and analysed via two‐tailed Student's *t*‐test. (D) Overexpressing IGSF10 suppressed A549 and H1299 cells proliferation measured by using Cell Counting Kit‐8. Data were presented as mean ± SD; the difference between control and IGSF10‐OE cells was analysed via two‐tailed Student's *t*‐test. (E) Overexpressing IGSF10 suppressed A549 and H1299 cells migration examined by using transwell chambers. Quantitative data were displayed on the side and presented as mean ± SD, the difference between control and IGSF10‐OE cells was analysed via two‐tailed Student's *t*‐test. (F) Overexpressing IGSF10 suppressed the tumorigenic capacity of A549 and H1299 cells. LUAD cells were infected with lentivirus which codes Flag‐tagged IGSF10 and then were injected subcutaneously into the dorsa of nude mice. Tumour weight was measured after 8 weeks. ns, non‐specific. *p < 0.05, **p < 0.01, ***p < 0.001.

The above findings suggested a possibility that IGSF10 specifically functions in LUAD cells. Pursuing this, exogenous IGSF10 was stably expressed in human LUAD cell line A549 and H1299 by viral infection to determine the impact of IGSF10 on LUAD cells in vitro (Figure 4A). As shown by the scratch assay, the migration of IGSF10‐overexpressed (henceforth referred to as “IGSF10‐OE”) A549 and H1299 cells was significantly slower than that of control cells (Figure 4B). To demonstrate whether overexpressing IGSF10 affects the LUAD cell survival and the ability of a single cell in developing a colony, we next performed a soft‐agar colony formation assay to assess the effect of IGSF10‐OE on A549 and H1299 cell survival and self‐renewal, and the data also testified to the reduced growth ability of IGSF10‐OE cells compared with control (Figure 4C). Meanwhile, IGSF10‐OE cells also showed a relatively lower proliferation rate than that of control cells (Figure 4D). Moreover, this result was further confirmed by a separate migration assay using Transwell chambers (Figure 4E). Likewise, further results from in vivo pulmonary tumorigenesis also proved that IGSF10‐OE cells possess much weaker tumorigenic capability than that of control cells (Figure 4F). Collectively, these results suggested that highly expressed IGSF10 can suppress the LUAD cell migration and growth, as well as the tumorigenic capacity.

### 3.5 IGSF10 Is Associated With Cell Proliferation and Ferroptosis Pathways in LUAD

**FIGURE 5:**
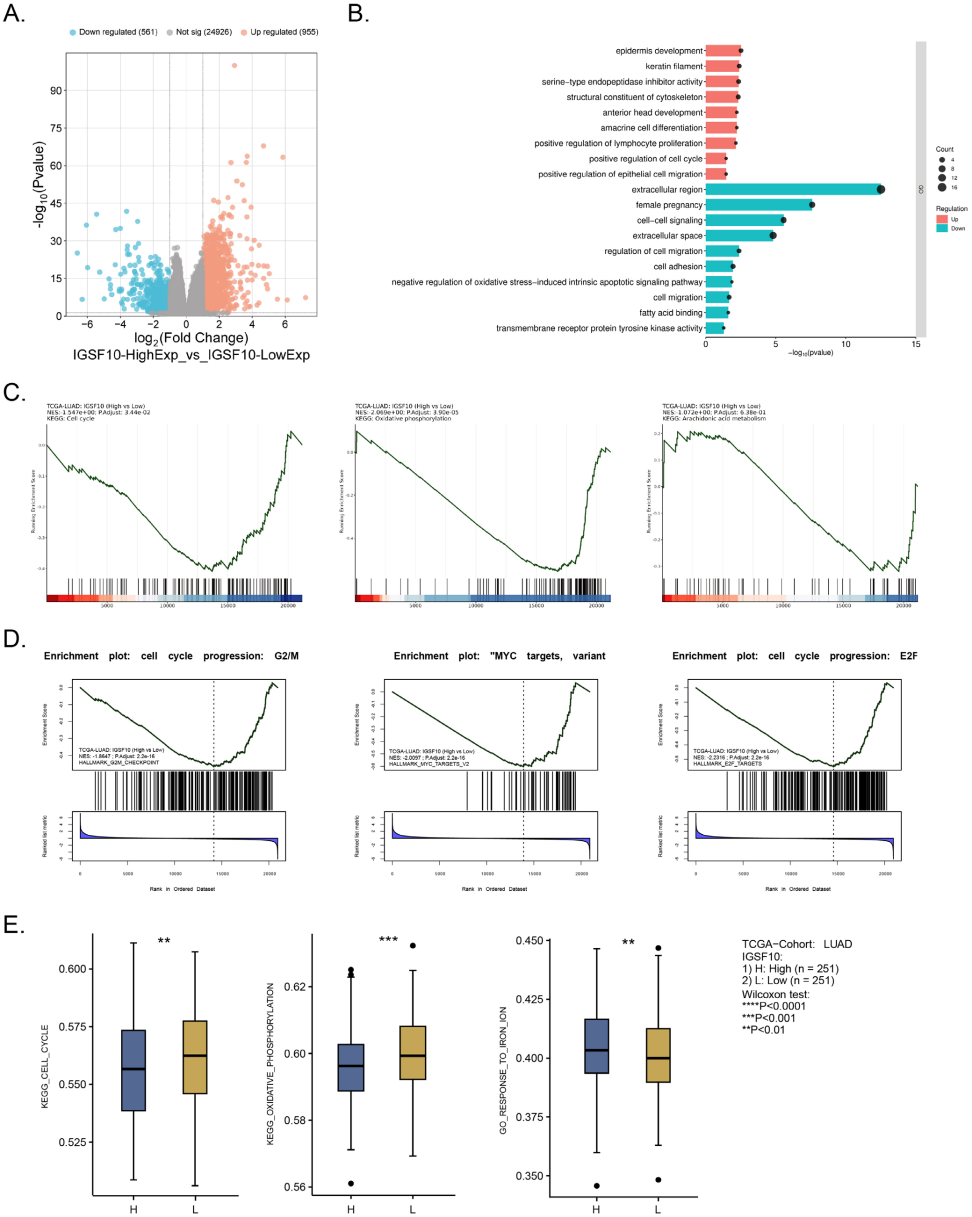
Differential identification of high and low expression IGSF10 and gene set enrichment analysis. (A) Volcano plot of mRNA expression changes between IGSF10 high‐ and low‐expressing LUAD samples. Red represents upregulation and blue represents downregulation. (B) Gene Ontology enrichment analysis plot of upregulated and downregulated genes. Blue represents downregulation, red represents upregulation, and dots represent the number of enriched genes. (C) GSEA comparing TCGA‐LUAD gene expression signature with high and low expression of IGSF10 using KEGG pathway gene set. (D) The HALLMARK dataset was used to analyse the GSEA pathway enrichment in IGSF10 high and low expression. (E) ssGSEA comparing TCGA‐LUAD gene expression signatures with high and low expression of IGSF10 using KEGG and GO gene sets. **p < 0.01, ***p < 0.001.

To understand the molecular mechanism of IGSF10's tumour‐promoting effect on LUAD, we analysed the differential gene expression between high and low expression samples of IGSF10 in the TCGA‐LUAD database using DESeq2, and we identified 955 significantly upregulated genes and 561 significantly downregulated genes (Figure 5A). Based on gene ontology (GO) enrichment analysis of upregulated and downregulated genes, our results showed that IGSF10‐related genes were mainly enriched in the processes of inhibiting cell migration, adhesion, and epithelial‐mesenchymal transition (EMT), as well as enhancing epithelial differentiation, which strongly supports its antitumor role in lung adenocarcinoma (Figure 5B). We further performed gene set enrichment analysis (GSEA) and single‐sample gene set enrichment analysis (ssGSEA) using the MSigDB key gene set (HALLMARK gene set) and the KEGG pathway gene set. GSEA analysis showed that the LUAD sample with high IGSF10 expression exhibited significant negative enrichment in the cell cycle (NES = −1.547, FDR = 0.034) and oxidative phosphorylation (NES = −2.069, FDR = 3.90 × 10^−5^), and negative enrichment in the arachidonic acid metabolism (Figure 5C), indicating that IGSF10 exerts a potent anti‐cancer effect by simultaneously inhibiting tumour cell proliferation and mitochondrial energy metabolism. We further analysed the enrichment of IGSF10 in the Hallmark gene set, and the results showed significant enrichment at the G2/M checkpoint (NES = −1.864, FDR = 2.2 × 10^−16^), E2F target genes (NES = −2.231, FDR = 2.2 × 10^−16^), and MYC target gene V2 (NES = −2.009, FDR = 2.2 × 10^−16^). This indicates that IGSF10 potently inhibits LUAD cell proliferation by simultaneously blocking G1/S and G2/M switching and antagonising MYC‐driven oncogenic transcriptional programs (Figure 5D). Further validation using ssGSEA showed that the activities of cell cycle (*p* < 0.01) and oxidative phosphorylation (*p* < 0.001) were significantly lower in LUAD patients with high IGSF10 expression than in the low expression group (Figure 5E). Furthermore, IGSF10 expression level was significantly positively correlated with the ferroptosis‐related pathway ‘Response to iron ion’ (*p* < 0.01), indicating that high IGSF10 expression activates this pathway. We have demonstrated, from multiple dimensions, that IGSF10 plays a comprehensive tumour‐suppressive role in LUAD, and also plays a role in the ferroptosis‐related pathway ‘Response to iron ion’ (*p* < 0.01). To determine whether decreased IGSF10 expression is part of a broader dysregulation of the immunoglobulin superfamily, we analysed the expression correlations of various IGSF members in the TCGA‐LUAD cohort. The results showed that IGSF10 was significantly negatively correlated with several immune‐related IGSF genes (Figures S1A–D), including the immunosuppressive molecules CD47 (*R* = −0.41, *p* < 0.001) and CD276 (*R* = −0.41, *p* < 0.001), as well as the tumour microenvironment regulators IGSF3 (*R* = −0.33, *p* < 0.001) and IGSF8 (*R* = −0.14, *p* < 0.001). These data suggest that low expression of IGSF10 and dysregulation of multiple IGSF family members may contribute to immunosuppression in the tumour microenvironment.

### 3.6 High Expression of IGSF10 Interferes With the Cell Cycle Transition of LUAD Cells

**FIGURE 6:**
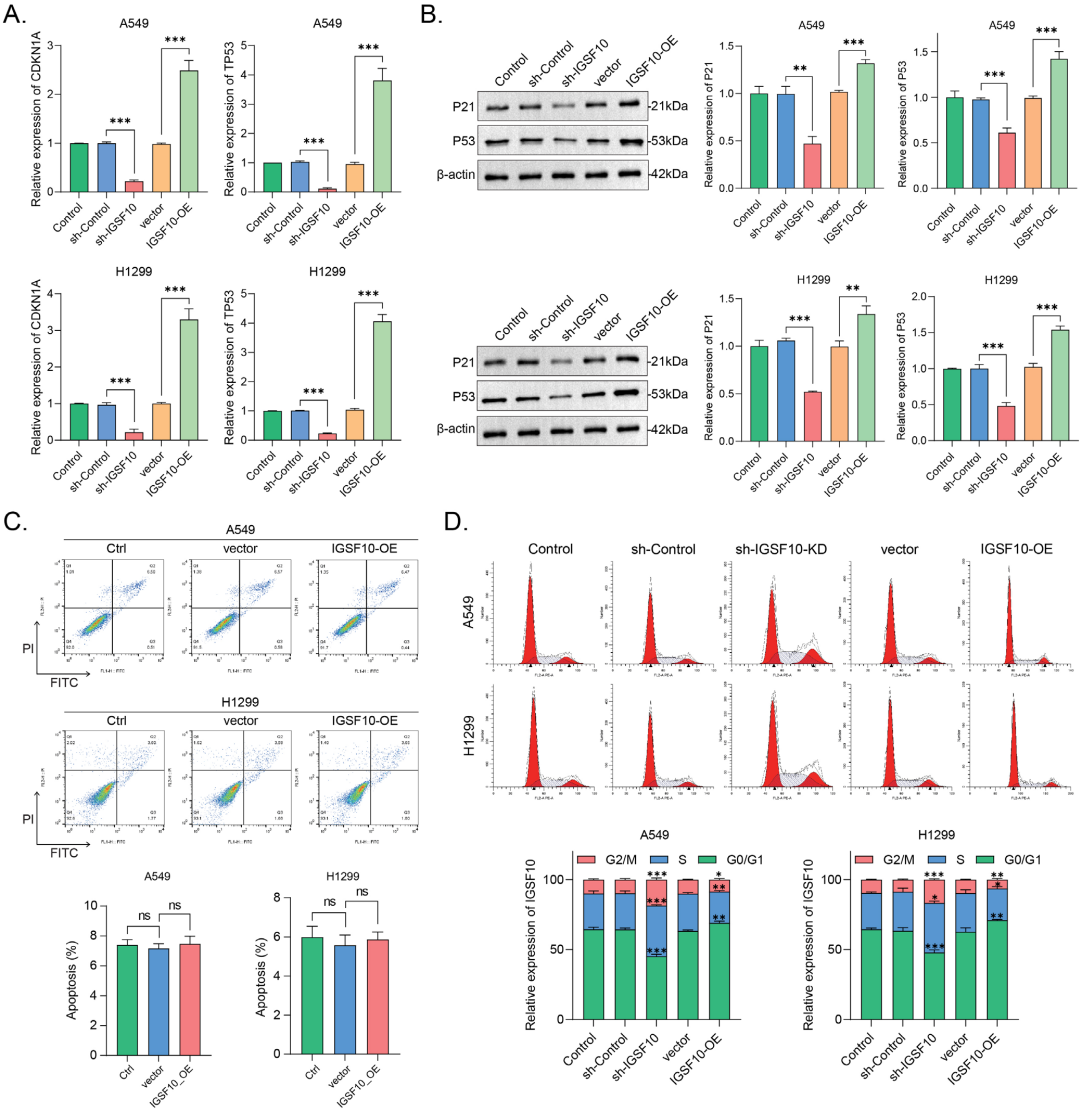
High expression of IGSF10 interferes with the cell cycle transition of LUAD cells. (A) The expression levels of CDKN1A and TP53 mRNA in A549 and H1299 cells overexpressing and knocked down IGSF10 were detected by qRT‐PCR. The results were analysed using a two‐tailed Student's *t*‐test. (B) Overexpressing and knocking down IGSF10 enhanced the expressions of p53 and p21 in A549 and H1299 cells examined by Western Blot. The quantitative histogram was displayed below and analysed via two‐tailed Student's *t*‐test. (C) Overexpressing IGSF10 caused little effect on the apoptosis of A549 and H1299 cells analysed by flow cytometry. Data were presented as mean ± SD. (D) Overexpressing and knocking down IGSF10 impeded the G_1_/S cell cycle transition of A549 and H1299 cells analysed by flow cytometry after PI staining. Data were presented as mean ± SD. ns, non‐specific. *p < 0.05, **p < 0.01, ***p < 0.001.

To investigate the mechanism by which IGSF10 regulates LUAD cell function, we first examined the expression levels of TP53 mRNA and its protein (p53) in real time. p53 is considered a key inhibitor in tumour development, with its main function being to induce apoptosis and cell cycle arrest in cancer cells [25]. As shown in Figure 6A,B, compared with the control group, the levels of TP53 mRNA and p53 protein were significantly increased in A549 and H1299 cells overexpressing IGSF10. However, IGSF10‐OE A549 and H1299 cells have presented a minimal apoptotic difference from the control cells (Figure 6C). Interestingly, we found that the cyclin‐dependent kinase (CDK) inhibitor p21 (also known as WAF1, CIP1, and CDKN1A), which was the first discovered transcriptional target to mediate p53‐induced G_1_ cell cycle arrest [26, 27, 28], was remarkably upregulated in IGSF10‐OE A549 and H1299 cells. The analysis of cell cycle progression using flow cytometry also showed that G_1_/S cell cycle transition was indeed impaired in IGSF10‐OE A549 and H1299 cells compared with the control cells. By contrast, knocking down IGSF10 in A549 and H1299 cells caused reductions of p53 and p21 while enhancement of G_1_/S cell cycle transition compared to that of control cells (Figure 6D), confirming that overexpression of IGSF10 can lead to a severe G_1_ cell cycle arrest via the p53‐p21 signaling axis in LUAD cells. To determine whether the enhanced tumour progression observed in IGSF10‐deficient cells is related to alterations in cell adhesion pathways, we examined the integrin β1/FAK pathway. Western blot analysis showed that integrin β1 protein levels were unchanged in both IGSF10‐overexpressing and IGSF10‐knockdown A549 and H1299 cells compared to controls. Interestingly, we found that p‐FAK was moderately but significantly reduced in IGSF10‐knockdown cells, while IGSF10 overexpression slightly increased p‐FAK protein levels (Figures S2A–D). These results suggest that IGSF10 may affect FAK activation without altering integrin β1 expression, but is unlikely to be the primary pathway driving phenotypic changes induced by IGSF10 deficiency.

### 3.7 High Expression of IGSF10 Inhibits the EMT of A549 and H1299 Cells via p53‐Triggering Ferroptosis

**FIGURE 7:**
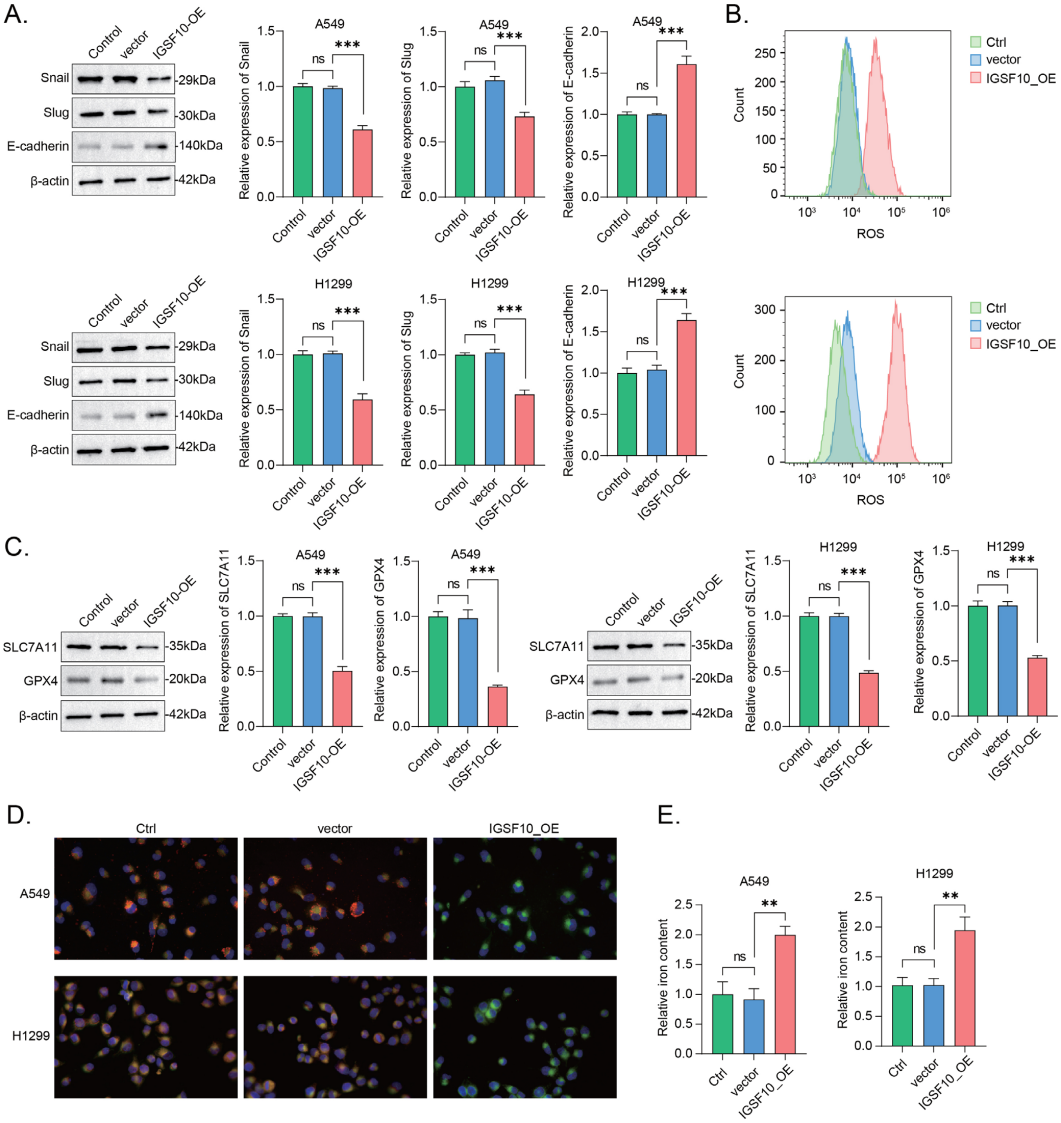
High expression of IGSF10 inhibits the EMT of A549 and H1299 cells via p53‐triggering ferroptosis. (A) Overexpressing IGSF10 inhibited the EMT of A549 and H1299 cells examined by detecting EMT on–off markers. The quantitative histogram was displayed on the side and analysed via two‐tailed Student's *t*‐test. (B) Overexpressing IGSF10 promoted the ferroptosis of A549 and H1299 cells examined by monitoring the cellular levels of ROS. (C) Overexpressing IGSF10 induced p53‐triggering ferroptosis in A549 and H1299 cells examined by detecting ferroptosis indicators. The quantitative histogram was displayed on the side and analysed via two‐tailed Student's *t*‐test. (D) Overexpressing IGSF10 promoted the ferroptosis of A549 and H1299 cells examined by monitoring the cellular levels of lipid peroxidation. (E) Overexpressing IGSF10 promoted the ferroptosis of A549 and H1299 cells examined by monitoring the cellular levels of iron, two‐tailed Student's *t*‐test. ns, non‐specific. **p < 0.01, ***p < 0.001.

**FIGURE 8:**
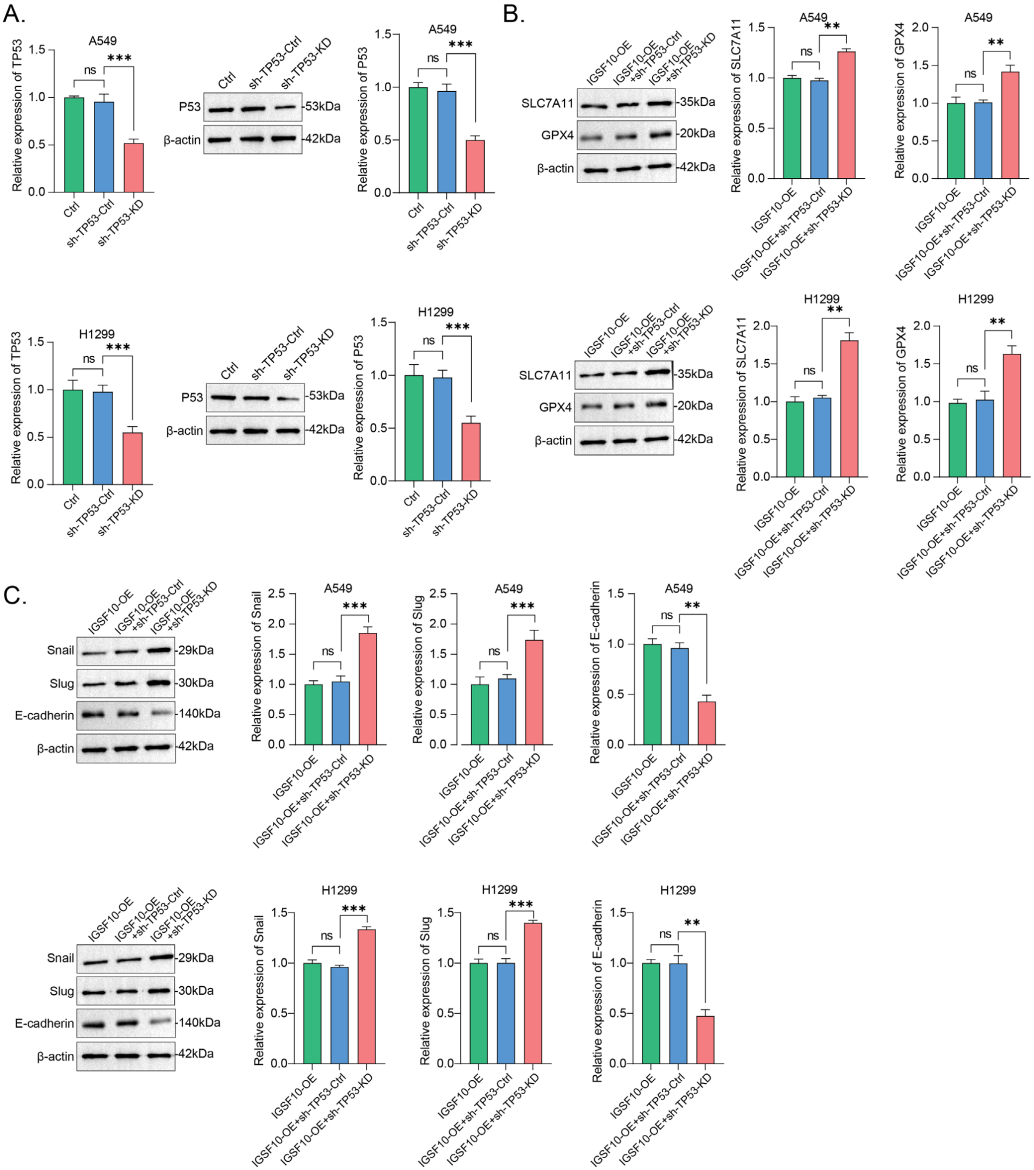
Low expression of p53 could significantly reverse the inhibitory effect of IGSF10 on EMT and ferroptosis. (A) Stable knockdown of Flag‐tagged TP53 in human adenocarcinoma cell A549 and H1299 was examined by qRT‐PCR and Western Blot analysis. (B) Knocking down TP53 reversed IGSF10‐induced p53‐triggering ferroptosis in A549 and H1299 cells examined by detecting ferroptosis indicators. The quantitative histogram was displayed on the side and analysed via two‐tailed Student's *t*‐test. (C) Knocking down TP53 promoted the EMT of A549 and H1299 cells examined by detecting EMT on–off markers. The quantitative histogram was displayed on the side and analysed via two‐tailed Student's *t*‐test. ns, non‐specific. **p < 0.01, ***p < 0.001.

LUAD is the most prevalent pathological subtype of NSCLC, characterised by a highly metastatic property due to the susceptibility of cancer cells to EMT (epithelial‐mesenchymal transition), a continuous process that is critical for embryonic morphogenesis by affecting cell migration [1, 29]. The previous results have shown that the IGSF10‐OE A549 and H1299 cells migrate significantly slower than the control cells; we therefore estimated the prospective EMT status of A549 and H1299 cells by detecting these canonical mediators and/or the EMT on–off markers, including Snail, Slug, and E‐cadherin. Accordingly, the results revealed an inhibitory role of IGSF10‐OE on the EMT of A549 and H1299 cells (Figure 7A). To further validate the role of IGSF10 in EMT regulation, we examined the expression of epithelial and stromal markers in IGSF10 knockdown A549 and H1299 cells. Western blot analysis showed that, compared with control cells, IGSF10‐silenced cells exhibited decreased E‐cadherin expression, while Snail and Slug expression were significantly increased (Figure S3A). These data indicate that IGSF10 deficiency promotes a portion of the EMT phenotype in LUAD cells, consistent with the inhibitory effect observed with IGSF10 overexpression. Ferroptosis is a novel regulatory non‐apoptotic cell death distinguished from these well‐known programmed cell deaths including apoptosis, autophagy, and necrosis. Additionally, except for such conventional functions in cell cycle arrest, senescence, and diverse programmed cell deaths, emerging evidence has also defined p53 as a key bidirectional regulator of ferroptosis by adjusting the metabolism of iron, lipids, glutathione peroxidase 4 (GPX4), reactive oxygen species (ROS), and amino acids via canonical or non‐canonical pathways [30]. Given the apparent induction of p53 by overexpressing IGSF10 in A549 and H1299 cells (Figure 7B), it raised a worthwhile question of whether IGSF10 overexpression has any role in p53‐triggering ferroptosis. In this regard, we showed that overexpressing IGSF10 significantly induced ferroptosis in A549 and H1299 cells, as evidenced by the remarkable decreases in ferroptosis indicators such as SLC7A11 (solute carrier family 7a member 11) and GPX4 (Figure 7C). Similarly, we examined the expression levels of core regulatory proteins of ferroptosis in LUAD cells with knockdown of IGSF10, and the results showed that the expression of GPX4 and SLC7A11 was significantly increased (Figure S3B). Moreover, this outcome was also validated by timely monitoring the cellular levels of ROS, lipid peroxidation, and iron, all of which were evidently enhanced in IGSF10‐OE A549 and H1299 cells compared with the control cells (Figure 7D,E). Moreover, we detected these EMT and ferroptosis markers in TP53‐KD A549 and H1299 cells (Figure 8A), the results indicated that the low expression of TP53 could significantly reverse the inhibitory effect of IGSF10 on EMT and ferroptosis (Figure 8B,C). Taken together, these results suggested that overexpressing IGSF10 in A549 and H1299 cells could strengthen p53‐triggering ferroptosis, leading to the inhibition of EMT.

## 4 Discussion

IGSF10 is essentially a member of the immunoglobulin superfamily embodying numerous proteins that play pivotal roles in the sophisticated immunological network, such as various antibodies, cell surface receptors, and major histocompatibility complex (MHC) class I/II molecules, etc. [8]. However, the focus of IGSF10 is not on its prospective correlation with the immunological system, for example, B cells, CD4^+^/CD8^+^ T cells, neutrophils, macrophages, and dendritic cells, but on the close implications in some developmental diseases and various cancers. Recently, a systematic pan‐cancer analysis using a variety of bioinformatic ways concludes that *IGSF10* could serve as a novel prognostic marker and attainable immunotherapy for several malignancies, including lung cancer [15]. However, the pathogenesis of lung cancer and the regulatory mechanisms of IGSF10 are still poorly understood. In the current study, we demonstrated low expression of IGSF10 in lung tumour tissues and demonstrated that low expression of IGSF10 was significantly associated with overall survival in lung cancer patients according to the TCGA‐LUAD database. Furthermore, we elucidated the precise impact of IGSF10 on the biological functions of lung cancer cells, that is, highly expressed IGSF10 can suppress lung cancer cell migration and growth, as well as tumorigenesis. Meanwhile, in LUAD, there was a significant negative correlation between IGSF10 and key immunosuppressive factors (CD47, CD276) and tumour microenvironment regulators (IGSF3, IGSF8) among IGSF family members, indicating that the downregulation of IGSF10 is part of the IGSF family network dysregulation, which collectively affects the tumour microenvironment and promotes tumour progression and immune escape. This suggests that in the future, we can combine IGSF10 restoration strategies with CD47/CD276 blockade to inhibit the progression of lung adenocarcinoma. Mechanistically, IGSF10 overexpression induced G_1_ cell cycle arrest via the p53‐p21 signaling axis, leading to reduced growth and tumorigenesis of lung cancer cells. Meanwhile, low expression of IGSF10 primarily promotes the progression of lung adenocarcinoma (LUAD) through cell cycle dysregulation mediated by the p53‐p21 axis, and secondarily through alterations in cell adhesion signaling pathways. This dual mechanism synergistically enhances tumour invasion and growth. Notably, we also innovatively proposed that IGSF10 exerts a potential effect on ferroptosis, a hot topic that functions in a variety of diseases, especially cancer therapy [31]. In essence, ferroptosis is an iron‐dependent, non‐apoptotic cell death process that comprises many unique features, including abnormal metabolism and subsequent accumulation of iron, intracellular accumulation of iron‐induced ROS, enhanced lipid metabolism and peroxidation, and impairment of the system X_c_^−^‐GSH‐GPX4 axis, etc. Studies have shown that corosolic acid inhibits EMT in lung cancer cells by promoting YAP‐mediated ferroptosis, and eriocitrin inhibits EMT in lung adenocarcinoma cells via triggering ferroptosis [32]. Accordingly, our present results have shown that overexpressing IGSF10 can inhibit the EMT of lung cancer cells via p53‐triggering ferroptosis. However, we are very aware of our shortcoming in the exploration of the mechanism by which IGSF10 contributes to this, and an in‐depth investigation based on the existing clues and proposed hypotheses needs to be sought after. Overall, our findings further clarified the role of IGSF10 in lung cancer cells and theoretically suggested new avenues for the presumable IGSF10‐targeting therapy.

## Author Contributions

**Lianyu Cheng:** software (equal), validation (equal), writing – original draft (equal). **Beibei Ma:** formal analysis (equal), validation (equal), visualization (equal). **Yun Zhao:** investigation (equal). **Chunyan Qin:** data curation (equal). **Lihe Jiang:** conceptualization (equal), methodology (equal), resources (equal), supervision (equal), writing – review and editing (equal). **Bo Ling:** conceptualization (equal), funding acquisition (equal), project administration (equal), supervision (equal), writing – review and editing (equal).

## Funding

This work was supported by: National Natural Science Foundation of China (82060540). Guangxi Natural Science Foundation (2025GXNSFHA069028). Zhejiang Key Laboratory of Diagnosis & Treatment Technology on Thoracic Oncology (Lung and Oesophagus) [grant number 2022K001]. The Grant of research project on high‐level talents of Youjiang Medical College for Nationalities (Grant No. YY2021SK02). Laboratory of Pollution Exposure and Health Intervention of Zhejiang Province (Grant No. 202300011).

## Ethics Statement

Under the protocol approved by the Institutional Review Board, informed consent was obtained from the patients or their guardians. Animal experiments were carried out in accordance with the guidelines of the Ethics Committee of Youjiang Medical College for Nationalities.

## Consent

All authors consent to submit this manuscript to the journal and confirm that it has not been published or submitted elsewhere.

## Conflicts of Interest

The authors declare no conflicts of interest.

## Supporting information


**Figure S1:** Correlation of IGSF10 with other IGSF members in lung adenocarcinoma. (A–D) Scatter plots show the expression correlation between IGSF10 and representative IGSF members (CD47, CD276, IGSF3, IGSF8) in the TCGA‐LUAD cohort. Expression values are expressed as log2 (TPM + 1). Pearson correlation coefficients (*R*) and corresponding *p*‐value are indicated in each figure.

**Figure S2:** Effects of IGSF10 on p‐FAK and integrin β1 protein expression in LUAD cells. Western blot was used to detect the protein expression levels of p‐FAK and integrin β1 in A549 (A, B) and H1299 (C, D) cells overexpressing and knocked down IGSF10. Two‐tailed Student's *t*‐test was used for analysis, ns: non‐specific, ***p < 0.001.

**Figure S3:** In LUAD cells with knockdown of IGSF10, the expression of EMT and ferroptosis regulatory proteins was significantly increased. (A) Western blot analysed the expression levels of EMT proteins (Snail, Slug, E‐cadherin) in A549 and H1299 cells with IGSF10 knockdown. The quantitative histogram was displayed on the side and analysed via two‐tailed Student's t‐test, ns: non‐specific, **p < 0.01, ***p < 0.001. (B) Western blot analysed the expression levels of ferroptosis core regulatory proteins (SLC7A11, GPX4) in A549 and H1299 cells with IGSF10 knockdown. The quantitative histogram was displayed on the side and analysed via two‐tailed Student's t‐test, ns: non‐specific, ***p < 0.001.

## Data Availability

The raw data and materials supporting this study are available from the corresponding author upon reasonable request.
