# Supplementary figures and images for: High Expression of IGSF10 Confers an Inhibitory Effect on the Progression of Lung Adenocarcinoma
Source: J Cell Mol Med. 2025 Dec 25;29(24):e70995. doi: 10.1111/jcmm.70995 (PMC12739995; doi:10.1111/jcmm.70995)

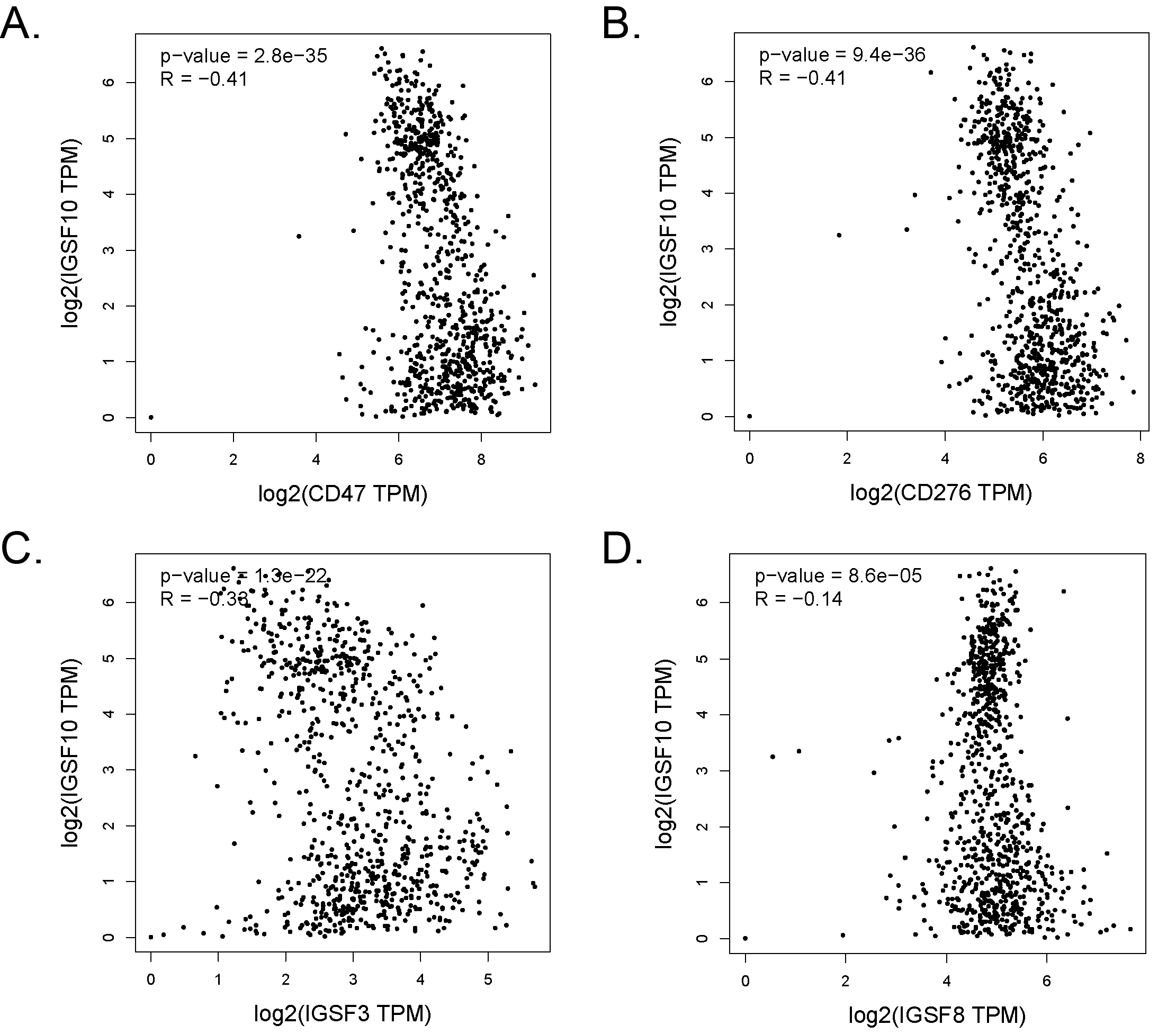

Supplement: Supplementary file 1 — Figure S1: Correlation of IGSF10 with other IGSF members in lung adenocarcinoma. (A–D) Scatter plots show the expression correlation between IGSF10 and representative IGSF members (CD47, CD276, IGSF3, IGSF8) in the TCGA‐LUAD cohort. Expression values are expressed as log2 (TPM + 1). Pearson correlation coefficients (R) and corresponding p‐value are indicated in each figure. [file JCMM-29-e70995-s002.jpg]

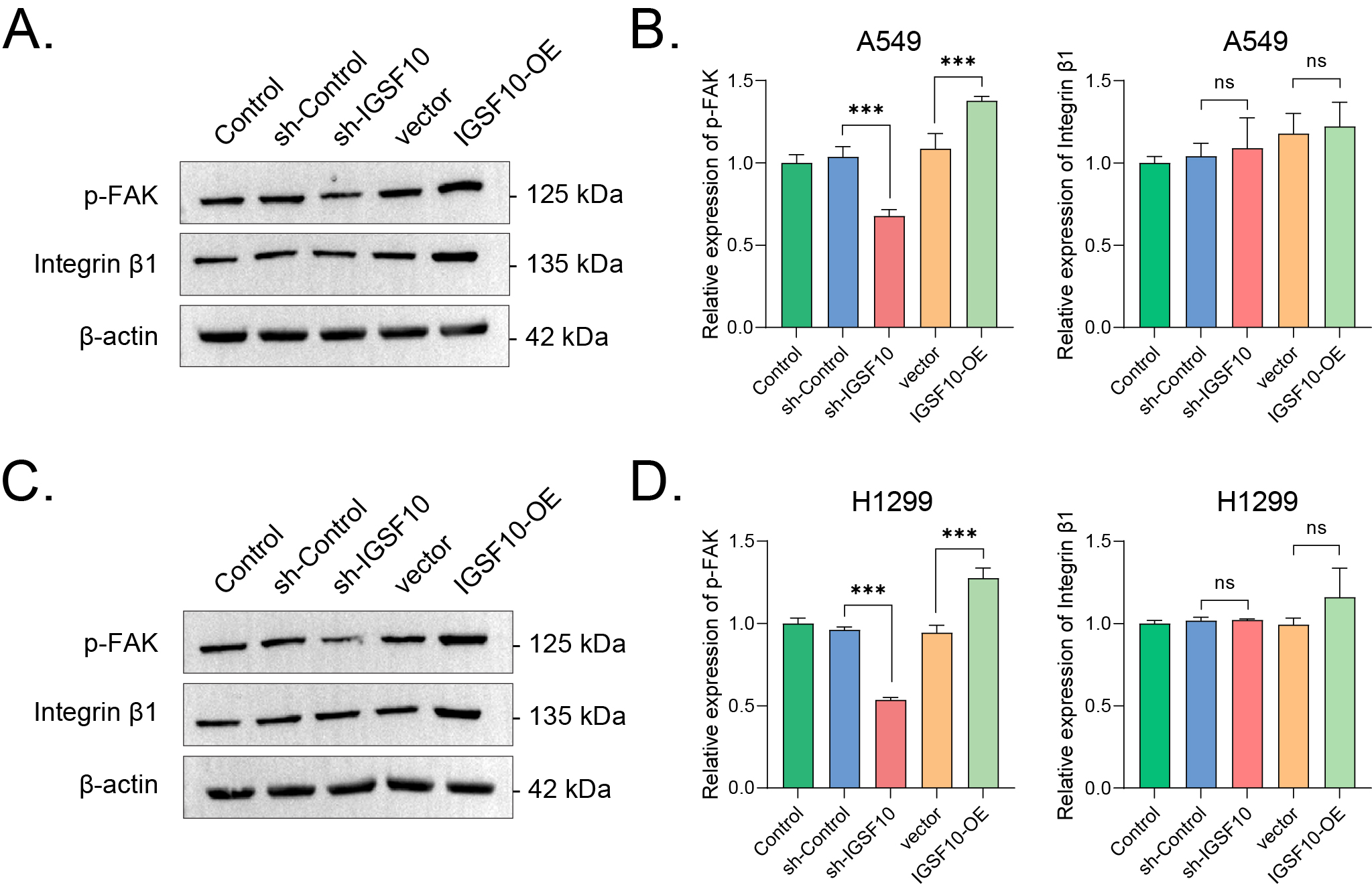

Supplement: Supplementary file 2 — Figure S2: Effects of IGSF10 on p‐FAK and integrin β1 protein expression in LUAD cells. Western blot was used to detect the protein expression levels of p‐FAK and integrin β1 in A549 (A, B) and H1299 (C, D) cells overexpressing and knocked down IGSF10. Two‐tailed Student's t‐test was used for analysis, ns: non‐specific, ***p < 0.001. [file JCMM-29-e70995-s001.jpg]

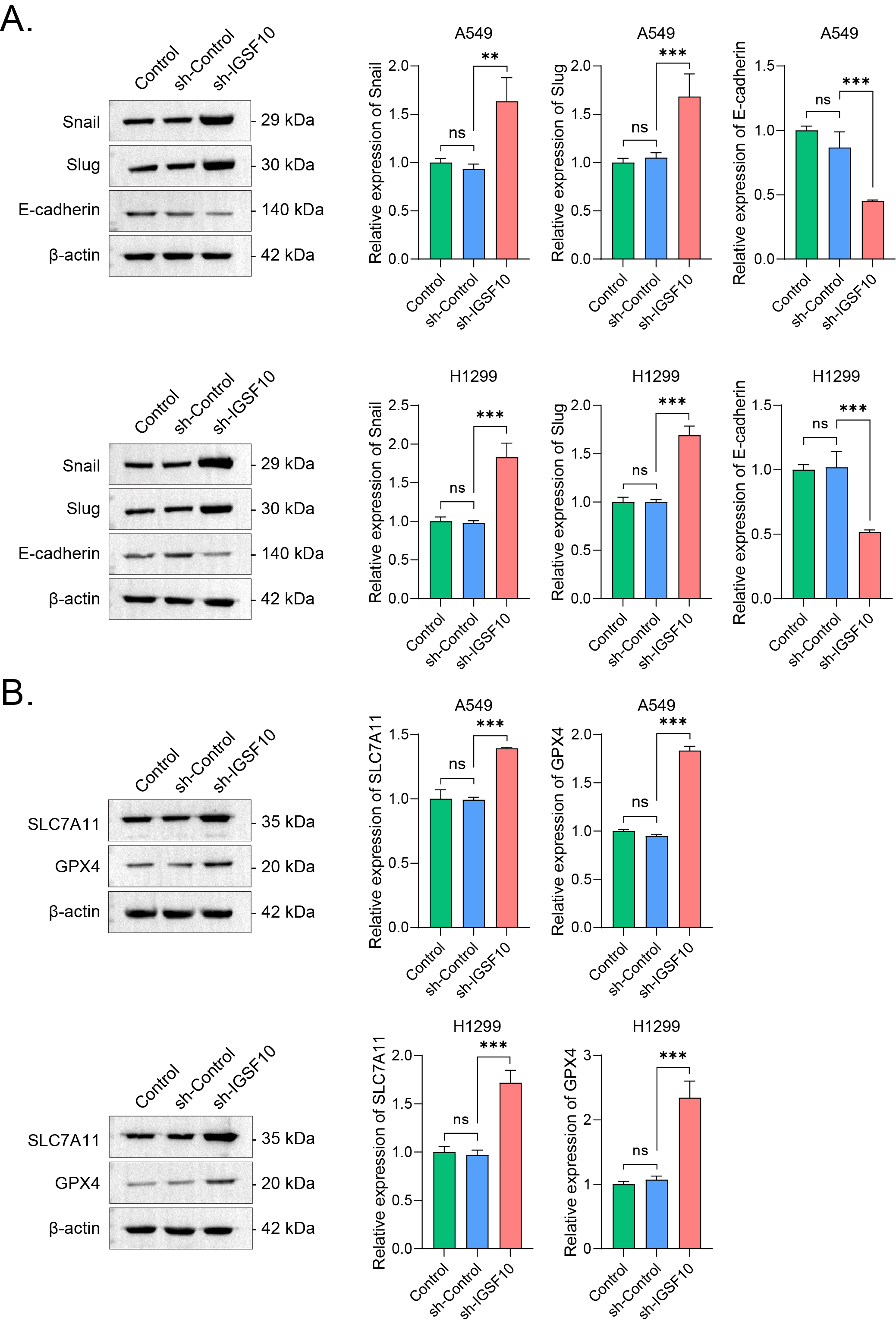

Supplement: Supplementary file 3 — Figure S3: In LUAD cells with knockdown of IGSF10, the expression of EMT and ferroptosis regulatory proteins was significantly increased. (A) Western blot analysed the expression levels of EMT proteins (Snail, Slug, E‐cadherin) in A549 and H1299 cells with IGSF10 knockdown. The quantitative histogram was displayed on the side and analysed via two‐tailed Student's t‐test, ns: non‐specific, **p < 0.01, ***p < 0.001. (B) Western blot analysed the expression levels of ferroptosis core regulatory proteins (SLC7A11, GPX4) in A549 and H1299 cells with IGSF10 knockdown. The quantitative histogram was displayed on the side and analysed via two‐tailed Student's t‐test, ns: non‐specific, ***p < 0.001. [file JCMM-29-e70995-s003.jpg]
